# Online Health Information Seeking for Mpox in Endemic and Nonendemic Countries: Google Trends Study

**DOI:** 10.2196/42710

**Published:** 2023-04-13

**Authors:** Thomas Shepherd, Michelle Robinson, Christian Mallen

**Affiliations:** 1 School of Medicine Keele University Staffordshire United Kingdom; 2 Research and Innovation Department St George’s Hospital Midlands Partnership NHS Foundation Trust Staffordshire United Kingdom

**Keywords:** monkeypox, mpox, infodemiology: surveillance, public health, health information seeking, Google Trends, joinpoint regression, epidemic, outbreak, infectious disease, disease, online

## Abstract

**Background:**

The recent global outbreak of mpox (monkeypox) has already been declared a public health emergency of international concern by the World Health Organization. Given the health, social, and economic impacts of the COVID-19 pandemic, there is understandable concern and anxiety around the emergence of another infectious disease—especially one about which little is known.

**Objective:**

We used Google Trends to explore online health information seeking patterns for mpox in endemic and nonendemic countries and investigated the impact of the publication of the first in-country case on internet search volume.

**Methods:**

Google Trends is a publicly accessible and free data source that aggregates worldwide Google search data. Google search data were used as a surrogate measure of online health information seeking for 178 days between February 18 and August 18, 2022. Searching data were downloaded across this time period for nonendemic countries with the highest case count (United States, Spain, Germany, United Kingdom, and France) and 5 endemic countries (Democratic Republic of Congo, Nigeria, Ghana, Central African Republic, and Cameroon). Joinpoint regression analysis was used to measure changes in searching trends for mpox preceding and following the announcement of the first human case.

**Results:**

Online health information seeking significantly increased after the publication of the first case in all the nonendemic countries—United States, Spain, Germany, United Kingdom, and France, as illustrated by significant joinpoint regression models. Joinpoint analysis revealed that models with 3 significant joinpoints were the most appropriate fit for these data, where the first joinpoint represents the initial rise in mpox searching trend, the second joinpoint reflects the start of the decrease in the mpox searching trend, and the third joinpoint represents searching trends’ return to searching levels prior to the first case announcement. Although this model was also found in 2 endemic countries (ie, Ghana and Nigeria), it was not found in Central African Republic, Democratic Republic of Congo, or Cameroon.

**Conclusions:**

Findings demonstrate a surge in online heath information seeking relating to mpox after the first in-country case was publicized in all the nonendemic countries and in Ghana and Nigeria among the endemic counties. The observed increases in mpox searching levels are characterized by sharp but short-lived periods of searching before steep declines back to levels observed prior to the publication of the first case. These findings emphasize the importance of the provision of accurate, relevant online public health information during disease outbreaks. However, online health information seeking behaviors only occur for a short time period, and the provision of accurate information needs to be timely in relation to the publication of new case-related information.

## Introduction

Mpox (monkeypox) is a viral zoonosis (transmitted to humans from animals) with symptoms similar to smallpox, although it is clinically less severe [1]. The current mpox outbreak was declared a public health emergency of international concern (PHEIC) by the World Health Organization (WHO) on July 23, 2022. As of September 13, 2022, there have been approximately 57,995 confirmed cases globally, with 511 reported in countries where cases have occurred previously and 57,484 confirmed cases in countries that previously never reported mpox infection [2]. The current outbreak of mpox virus in humans suggests biological changes or changes in human behavior with these changes being precipitated by reduced smallpox immunity, relaxation of COVID-19 transmission prevention measures, recommencement of international travel, and increased sexual interactions associated with large gatherings [1,3]. Although human-human transmission was previously thought to be rare, it is attributed to respiratory droplets or direct contact with mucocutaneous lesions of an infected individual [4]. Given the health, social, and economic impacts of the COVID-19 pandemic, there is understandable concern and anxiety around the emergence of another infectious disease [5].

The growth of global internet usage has made health-related information more accessible [6]. Individuals increasingly use the internet to search for health-related information. The data generated by this search traffic provide a rich data set to monitor health information seeking behaviors [7] and can be used as a “surrogate” measure of disease awareness [8]. Using Google Trends, a free and publicly accessible tool, it is possible to access such internet traffic data. Google Trends analyzes Google searches, generating data on the geographical and temporal search patterns according to specified keywords [9]. Google Trends determines the proportion of searches for a user-specified search term among all searches performed with Google. It uses these data to provide a relative search volume (RSV), which is the query share of a particular term for a given location and time period, normalized by the highest query share of that search term [7]. Utility of these data is still in its infancy, but the data have been used successfully to predict outbreaks of influenza [10], norovirus [11], and COVID-19 [12]; the impact of disease awareness programs [13,14]; and the seasonality of searching for pain-related conditions [15]. With mpox being a rare disease and relatively unknown outside of endemic regions, we aimed to explore online health information seeking behaviors in both endemic and nonendemic regions using Google Trends. We hypothesized that RSV would be low for mpox until case notifications were publicized in the media, which would precipitate an initial surge in mpox searching before a sharp decline to previously observed searching levels, replicating health information searching patterns seen in our previous work [13,14] and highlighting the increasing public awareness of this infection.

## Methods

### Google Trends

Google Trends data come from a sample of the total Google search data, which are categorized, connected to a topic, and anonymized. Searches with special characters, those with a very low search volume, and repeated searches from the same individuals over a short period are excluded. Each sampled data point is then scaled to the total number of searches done over the selected location and time period. This “relative popularity” is given in the form of an RSV as a value between 0 and 100 (8). Google Trends data only reflect Google searches initiated by the user and not the subsequent online activity in response to the findings of the initial search.

### Mpox Case Data

Mpox case data were recorded on August 18, 2022, and were accessed via the Centers for Disease Control and Prevention (CDC) 2022 Mpox Outbreak Map [2]. The CDC global map displays case data per country, where cases are confirmed by laboratory testing. The 5 nonendemic countries with the highest case numbers at the time of accessing the data were the United States (13,516), Spain (5792), Germany (3213), United Kingdom (3081), and France (2749). For endemic countries, cases were highest for the Democratic Republic of Congo (163), Nigeria (157), Ghana (47), Central African Republic (8), and Cameroon (7). Ghana was included in the endemic countries list; although not reporting confirmed cases until the 2022 outbreak, the country was identified as the source of a shipment that led to a 2003 outbreak [2]. Internet penetrance (as a percentage of the total population) for the countries in the study were as follows: United States (91.2%), Spain (89.3%), Germany (93.3%), United Kingdom (96.6%), France (92.2%), Democratic Republic of Congo (7.3%), Nigeria (63.8%), Ghana (45.9%), Central African Republic (3.7%), and Cameroon (20.5%) [16].

### Google Trends Reporting

Users can manipulate aspects of Google Trends to tailor their search. To ensure transparency, reproducibility, and quality of our methods, we followed the reporting guidelines recommended by Nuti et al [7].

### Search Input

The term “monkeypox” was used to facilitate trend searching with “topic” instead of “search term.” Searches and data downloads were completed before the WHO recommendation to use the term mpox (November 28, 2022). Google describes a topic as “a group of terms that share the same concept in any language.” The example they provide is that searching for “London” as a topic will yield results for searches including “capital of the UK” and “Londres,” the Spanish name for London [17]. The topic feature encompasses searches for relevant subthemes. For instance, our search input “Monkeypox” will have included Google Trends data for the search input “Monkeypox symptoms.” The topic feature encompasses linguistic variations of the search input. This is especially important as we analyzed data from countries with differing official languages. Accommodating for linguistic variations enabled us to measure search inputs in other languages used within countries besides the official language, allowing for greater representation of searching behaviors across countries.

### Search Variables

Data were accessed from Google Trends on August 18,2022. Daily RSV was downloaded for each country over a 6-month period to cover the start of the outbreak to the time of writing. Daily RSV was therefore downloaded for a period of 178 days between February 18, 2022, and August 18, 2022.

### Analytic Method

A time trend analysis was carried out on the RSV data as an indicator of health information seeking behaviors leading up to, and following, the announcement of the first human case. The joinpoint regression model was used to identify points where statistically significant changes in the linear slope of the trend had occurred. These best-fitting points, called “joinpoints,” mark a statistically significant increase or decrease in RSV. The Joinpoint Regression Programme (version 4.6.0.0) was used to undertake the analysis [18]. This statistical software quantitatively identifies time points in which a temporal trend significantly changes and estimates the regression function with previously identified joinpoints [19]. In light of our previous Google Trends work [13,14], the analysis was preset with criteria to find a minimum of 0 and a maximum of 3 joinpoints. This was to capture an initial increase in RSV when initial mpox cases were discovered and publicized in the country, a second joinpoint for when searching would typically fall following this initial increase, and a third joinpoint for when the downturn resumes back to levels seen prior to the publication of the first case. The model selection method was a permutation test, testing for an overall significance level at .05.

## Results

### Nonendemic Regions

#### United States

**Figure 1:**
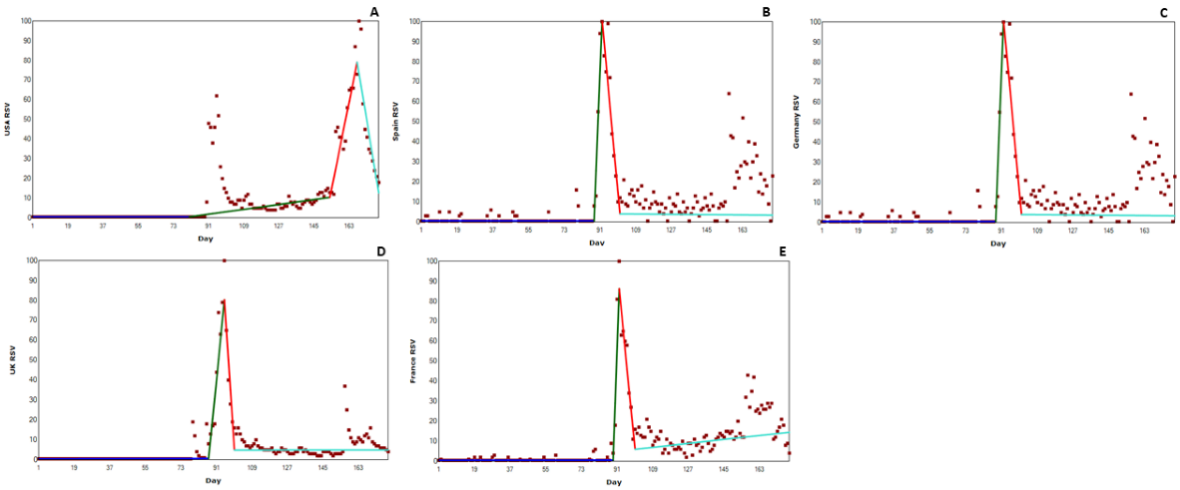
Changes in Relative Search Volume (RSV) for mpox (monkeypox) in nonendemic countries. Each data point indicates the RSV measured on the specified day. RSV is the query share of a particular term for a given location and time period, normalized by the highest query share of that search term. Color scheme: blue = first slope; green = second slope; red = third slope; and mint green = fourth slope. Number of slopes present depends on the number of joinpoints identified. Joinpoints mark a statistically significant change in the linear slope of the trend in the studied time period. (A) RSV data for United States. (B) RSV data for Spain. (C) RSV data for Germany. (D) RSV data for United Kingdom. (E) RSV data for France.

RSV data are presented in Figure 1A. Analysis showed 3 significant joinpoints (*P*<.001). There was a spike in searching around the date of the first case (May 18, 2022, or day 89). This increase in RSV was the first joinpoint (*P*<.001). The most significant peak and the most appropriate joinpoint model was applied to the time that mpox was declared a PHEIC by the WHO on July 23, 2022, or day 155; this increase in RSV of approximately 800% represented the second joinpoint (*P*<.001). Searching peaked at day 167 (14 days after the steep rise in searching began); the following reduction in searching represented the third significant joinpoint (*P*<.001), back to pre-PHEIC announcement levels.

#### Spain

RSV data for Spain are presented in Figure 1B. Analysis revealed 3 significant joinpoints (*P*<.001). The first mpox case in Spain was reported on May 18, 2022, or day 89. The first significant joinpoint (*P*=.03) was found on day 88, which led to a significant, sharp rise in RSV of approximately 1000% in 4 days to the peak on day 92. Day 92 was also the location of the second significant joinpoint (*P*=.002), which preceded a sharp decline in RSV for 9 days until joinpoint 3 (*P*<.001) on day 101, where RSV clustered around a level of 10.

#### Germany

RSV data for Germany is presented in Figure 1C. Analysis showed 3 significant joinpoints (*P*<.001). The first significant joinpoint (day 88) led to an approximate 1250% increase in searching. The first case in Germany was reported on May 21, 2022, or day 91. RSV peaked at day 92, representing the second joinpoint (*P*=.03), with the steep decline in searching reaching the third significant joinpoint (*P*<.001) 9 days later. Despite the steep reduction in searching after the initial peak, RSV has remained higher than prior to the first case announcement.

#### United Kingdom

A significant 3 joinpoint model was found for United Kingdom RSV data (*P*<.001) and is presented in Figure 1D. The first case was on May 6, 2022, or day 63; although this led to an increase in RSV, the first significant joinpoint was found on day 87 (*P*<.001), reflecting an approximate 444% increase over an 8-day period. The second significant joinpoint (*P*<.001) was the RSV peak (day 95), precipitating a steep fall over the next 5 days, where the third significant joinpoint is found (*P*<.001). RSV from this joinpoint onward is only marginally higher than prior to the first case.

#### France

RSV data from France are presented in Figure 1E. Analysis revealed 3 significant joinpoints (*P*<.001). The first case was on May 19, 2022, or day 90. The first significant joinpoint was found on day 89 (*P*<.001), preceding the steep and immediate rise of approximately 2000% to the peak RSV, 3 days later on day 92. Day 92 was also the second significant joinpoint (*P*=.02). The following 8 days reflected a steep decline in RSV from the peak down to levels shown at joinpoint 1. Joinpoint 3 was reported on day 100 (*P*=.01), although there had since been a steady increase of approximately 100% in RSV.

### Endemic Countries

#### Democratic Republic of Congo

**Figure 2:**
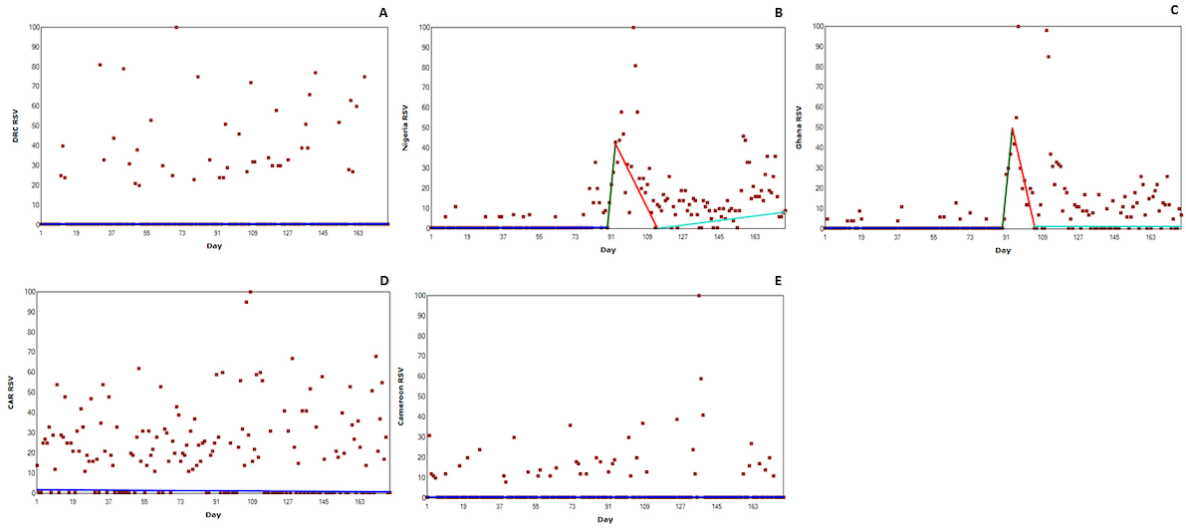
Changes in Relative Search Volume (RSV) for mpox (monkeypox) in endemic countries. Each data point indicates the RSV measured on the specified day. RSV is the query share of a particular term for a given location and time period, normalized by the highest query share of that search term. Color scheme: blue = first slope; green = second slope; red = third slope; and mint green = fourth slope. Number of slopes present depends on the number of joinpoints identified. Joinpoints mark a statistically significant change in the linear slope of the trend in the studied time period. (A) RSV data for Democratic Republic of Congo. (B) RSV data for Nigeria. (C) RSV data for Ghana. (D) RSV data for Central African Republic. (E) RSV data for Cameroon.

Mpox RSV data for the Democratic Republic of Congo is presented in Figure 2A. No significant joinpoints were found during analysis (*P*=.92). RSV levels range from 0 to 100, with a mean of 10.69; however, as illustrated, RSV data points were widely spread.

#### Nigeria

Figure 2B displays RSV data for Nigeria. A significant 3 joinpoint model was found in the analysis (*P*<.001). Joinpoint 1 was found on day 89, before a 1000% increase in RSV to the peak on day 99. The second significant joinpoint was found earlier, on day 93 (*P*<.001), precipitating a less steep decline in RSV seen elsewhere. RSV post peak was much higher and varied after the third significant joinpoint found on day 114 (*P*=.002); it shows searching gradually increased toward day 178.

#### Ghana

RSV data for Ghana is presented in Figure 2C. A significant 3 joinpoint model was reported for RSV data in Ghana (*P*<.001). The first significant joinpoint (*P*=.03) was found on day 89, preceding the first case reported in Ghana on June 8, 2022, or day 110. The second significant joinpoint was reported on day 94 (*P*=.003) at an RSV of 50 (50% of the peak RSV found on day 97). The third significant joinpoint (*P*<.001) was found on day 105 once the RSV had declined. Post joinpoint 3, RSV gradually increased toward the end of the study period.

#### Central African Republic

RSV data for Central African Republic is presented in Figure 2D. Analysis revealed no significant joinpoints in the data (*P*=.43). RSV levels ranged from 0 to 100 with a mean of 19.87; however, no discernible trends were observed in the RSV data.

#### Cameroon

RSV data for Cameroon is presented in Figure 2E. No significant joinpoints were found in the analysis (*P=*.94). RSV levels ranged from 0 to 100 with a mean of 5.92. RSV remained relatively consistent throughout the study period, without observable trends.

## Discussion

This study investigated online health information seeking behaviors for mpox in both endemic and nonendemic countries as a result of the 2022 global outbreak. We used Google Trends data as a surrogate for online health information seeking and joinpoint analysis software to analyze the data.

Data predominantly reflected the same pattern, whereby the first human case of mpox triggered a surge in online health information seeking. This pattern was observed for Spain, Germany, United Kingdom, France, Nigeria, and Ghana. Although a significant 3 joinpoint model was found in the RSV data from the United States, the declaration of the mpox outbreak as a PHEIC led to a more significant peak compared to the publication of the first case. Furthermore, the 3 significant joinpoint pattern was not observed in Central African Republic, Democratic Republic of Congo, or Cameroon—despite Democratic Republic of Congo being the African country with the current highest number of cases. However, our data suggested that although the RSV for mpox was active in the country, the levels of searching were more sporadic and seemingly not following any discernible trend. Additionally, these 3 countries also had much lower internet penetrance (Democratic Republic of Congo: 7.3%; Central African Republic: 3.7%; and Cameroon: 20.5%) compared to the other African countries (Nigeria: 63.8% and Ghana: 45.9%).

When a significant steep rise in online searching was reported (in both nonendemic and previously endemic countries), the trends all reflected the same type of pattern. A steep increase in searching behavior that peaks quickly, a peak that is rarely sustained for more than a few days, before searching levels return to those seen prior to the initial surge. This is consistent with previous work exploring the impact of the disease-specific awareness days on health-related information searching [13,14]. This observation can be attributed to several factors. First, Google Trends data only accounts for one element of online health information searching, with other searching and subsequent learning taking place on other websites or platforms, including social media. Mahabir et al [20] proposed a stimulus-awareness-activism framework, in which an individual’s awareness leads to both further online and offline activity related to the topic. Mahroum et al [21] assessed the digital behaviors in response to a Chikungunya outbreak by analyzing the interplay between novel data streams, such as website searches or social networks [21]. Google Trends was found to positively affect twitter activity. Essentially, users tended to search for “Chikungunya” on Google in response to notified cases, and then, interacted with the topic on Twitter.

Further awareness can be acquired through browsing key topic websites. Users may find these through Google and thus subsequently directly access and use them as a source of health information. For instance, Kranenburg et al [22] explored Google Trends data for “blood donation” as a result of World Blood Donor Day, reporting that national-level blood bank websites were visited twice as much as they would be typically, and this positively correlated with the RSV for “blood donation.” In light of this, it is possible that the initial surge in online health information seeking lasts longer than our data might suggest, manifested though other data streams. Future studies should assess the relationships between traffic to disease-specific websites or information sources and relevant Google RSVs.

Publication of first cases of new or emerging diseases clearly promotes a surge in online health information seeking, especially if little is known about that condition. The spike in searching behaviors using Google occurs in a relatively short window, which has significant implications for the timely provision of accurate and evidence-based public health–related information, around methods of infection, transmission prevention methods, and symptoms. As seen in the RSV from the United States, other events such as declaring a disease outbreak as PHEIC can lead to exponential increases in searching (although the surge period in information seeking is still short). Regardless, health bodies and governmental organizations must work to ensure that this information is searchable and in place (where it exists) as new disease cases or large news publication events are disclosed to the public. Conversely, this short window of public health information seeking is when harmful misinformation can also be found and become embedded in public consciousness.

In countries where no significant trends in online health information seeking were found—particular endemic regions (eg, Democratic Republic of Congo, Central African Republic, and Cameroon)—RSV remained relatively constant throughout the study period, and that reinforces the need for high-quality disease information to be publicly accessible. As internet penetrance is low in these countries, the dissemination of public health–related information (eg, transmission prevention measures) must rely on different traditional infrastructures and media (eg, newspapers and radio). Further work is required to explore potential seasonal or climate-related factors on mpox searching trends in these countries. Research is also required on the effectiveness of the dissemination of public health information using traditional media and especially on how this information is interpreted.

There are limitations with using internet search query data and Google Trends data specifically as a measure of online health information seeking. First, only those with internet access can be accounted for in online health information seeking data; therefore, our findings are only valid for health information seeking that takes place online. Internet penetrance was high in the nonendemic countries but substantially lower in the endemic countries, and that could have influenced our results [16]. However, a study on the worldwide Zika-related digital behavior [23] found that activity came mainly from the Central and South America region, even though the Zika outbreak breached beyond this region and received global news coverage. Second, the observed interest level is limited to those who use Google as a search engine. However, in the studied time period, Google represented 95.6% of the search engine market share in Africa [24], indicating a sufficient level of internet access and use of Google [9]. Additionally, the calculation of the RSV is dependent on mathematical assumptions and approximations, which are not public. However, previous evidence suggests trends have been accurate in approximating the seasonality of conditions [15,25] and at predicting influenza outbreaks, comparable to the US CDC health surveillance [10]. Other factors may also contribute to the increase in online health information seeking behaviors observed in this study, such as general mpox news or the reporting of international mpox cases. A systematic review on the use of Google Trends in health-related research [7] revealed poor documentation of the methodology in most studies, limiting reproducibility of study findings. We have adhered to their documentation recommendations to ensure transparency and reproducibility of our methodology, allowing for potential comparisons of findings over time.

In conclusion, we explored online health information seeking behaviors for mpox in endemic and nonendemic countries during the 2022 outbreak. We observed a large spike in searching for mpox-related information after the announcement of the first case in the nonendemic countries and in Ghana and Nigeria among the endemic countries. Consistent with our previous work, we found that this increase in searching was only for a short period, which has significant implications for the timely provision of accurate and accessible public health–related information on the web to increase public understanding of new diseases and reduce the likelihood of the spread of misinformation.

## Abbreviations

CDC: Centers for Disease Control and Prevention
PHEIC: public health emergency of international concern
RSV: relative search volume
WHO: World Health Organization

## Data Availability

The data sets generated and analyzed during this study are available from the corresponding author on reasonable request.
